# Feasibility, Usability, and Implementation Context of an Internet-Based Pain Education and Exercise Program for Chronic Musculoskeletal Pain: Pilot Trial of the ReabilitaDOR Program

**DOI:** 10.2196/35743

**Published:** 2022-08-30

**Authors:** Iuri Fioratti, Gisela Cristiane Miyamoto, Junior Vitorino Fandim, Camila Pereira Pontes Ribeiro, Geovana Domingues Batista, Gabriella Evangelista Freitas, Andressa Santos Palomo, Felipe José Jandré dos Reis, Leonardo Oliveira Pena Costa, Christopher G Maher, Bruno Tirotti Saragiotto

**Affiliations:** 1 Masters and Doctoral Programs in Physical Therapy Universidade Cidade de São Paulo São Paulo Brazil; 2 Department of Health Sciences, Faculty of Science Vrije Universiteit Amsterdam Amsterdam Public Health Amsterdam Netherlands; 3 Physical Therapy Department Instituto Federal do Rio de Janeiro Rio de Janeiro Brazil; 4 Postgraduation Program, Clinical Medicine Department Universidade Federal do Rio de Janeiro Rio de Janeiro Brazil; 5 Pain in Motion Research Group, Department of Physiotherapy, Human Physiology and Anatomy, Faculty of Physical Education & Physiotherapy Vrije Universiteit Brussel Brussels Belgium; 6 Institute for Musculoskeletal Health The University of Sydney Sydney Australia

**Keywords:** telerehabilitation, musculoskeletal pain, implementation science, feasibility study, chronic pain, pain, pilot study, eHealth, exercise, telehealth, self-management

## Abstract

**Background:**

Internet-based self-management programs and telerehabilitation initiatives have increased and have been extensively used for delivering health care in many areas. These programs overcome common barriers that patients face with traditional face-to-face health care, such as travel expenditures, lack of time, and high demand on the public health system. During the COVID-19 pandemic, this mode of web-based health care delivery had become more popular. However, there is still a lack of studies testing this mode of delivery in low- and middle-income countries. To gain a better understanding of the context, feasibility, and factors involved in the implementation of a web-based program, pilot and implementation studies are necessary. These studies can better inform whether a strategy is feasible, acceptable, and adequate for its purposes and for optimizing resource allocation.

**Objective:**

This study aims to evaluate the feasibility, usability, and implementation context of a self-management internet-based program based on exercises and pain education (ReabilitaDOR) in people with chronic musculoskeletal pain and to compare this program with a program using only a web-based self-management booklet.

**Methods:**

The study design was a parallel pilot study of a prospectively registered, assessor-blinded, 2-arm randomized controlled trial with economic evaluation. This study was performed using waiting lists of physiotherapy and rehabilitation centers and advertisements on social media networks. The participants were 65 patients with chronic musculoskeletal pain aged between 18 and 60 years. The effects of an 8-week telerehabilitation program based on exercises and pain education (intervention group) were compared with those of a program based only on a web-based self-management booklet (control group). The main outcome measures were implementation outcomes of patients’ perceptions of acceptability, appropriateness, feasibility, and usability of the program and the societal costs and feasibility of the main trial at 8-week posttreatment follow-up. Adverse events were also analyzed.

**Results:**

In total, 56 participants were analyzed at the 8-week follow-up. The intervention group showed responses with a mean of 4.5 (SD 0.6) points for acceptability, 4.5 (SD 0.5) points for appropriateness, and 4.5 (SD 0.6) points for feasibility measured on a 1 to 5 scale. All patients in the intervention group showed satisfactory responses to the system usability outcome. There is satisfactory evidence for the feasibility of the main trial. For costs related to the interventions, health care, patients, and loss of productivity at 8 weeks, we found a total expenditure of US $278.30 per patient in the intervention group and US $141.52 per patient in the control group. No adverse events were reported during the intervention period.

**Conclusions:**

We found that the ReabilitaDOR program is feasible, appropriate, and acceptable from the users’ implementation perspective. This system was considered usable by all the participants, and the main trial seemed feasible. Cost data were viable to be collected, and the program is likely to be safe.

**Trial Registration:**

ClinicalTrials.gov NCT04274439; https://clinicaltrials.gov/ct2/show/NCT04274439

## Introduction

Chronic pain is broadly defined as pain that persists for more than 3 months [1,2]. The most common chronic pain conditions are back and neck pain and knee and hip osteoarthritis, which are part of a broader group often called as chronic musculoskeletal pain [3-5]. Recent literature classifies chronic musculoskeletal pain as conditions characterized by persistent inflammation of infectious, autoimmune, or metabolic etiology [1]. This is a group of pain conditions that affects millions of people around the world and is responsible for an enormous economic burden, which impacts health services globally [5,6]. Chronic musculoskeletal pain is also the major cause of years lived with disability globally [5].

The management of chronic musculoskeletal pain is primarily multimodal, with noninvasive and nonpharmacological therapies as first-line options. These therapies include exercise, pain education, psychological, and physical therapies [7-11]. Although these recommendations from clinical practice guidelines are clear and usually do not involve complex programs [12,13], many people do not have access to adequate and affordable treatments. This can be due to geographical barriers (eg, people living in rural areas or Indigenous communities) or health care systems that may be overstretched with no capacity to provide timely and equitable access to health services [14]. Thus, there is a need for the development of remote strategies such as telerehabilitation [14,15] to improve access to health care.

Telerehabilitation is defined as the use of remote mechanisms and technologies for screening, diagnosis, education, treatment, and monitoring of a given condition [15,16]. Telerehabilitation can be delivered through telephone calls, smartphone apps, websites, or digital platforms that guarantee the population’s access to multimodal treatment strategies for chronic pain [14,15]. The use of telerehabilitation strategies has grown exponentially in the past years; however, the great majority of clinical trials and implementation studies of telerehabilitation are still being conducted in high-income countries [17]. Hence, there is a clear need for more clinical studies assessing telerehabilitation in low- and middle-income countries [14,17].

One important step before conducting a large clinical trial is to test the feasibility of the process involved in the main study, such as recruitment rates and the resources needed [18-20]. Trials using web-based interventions may also need to test the user’s usability of the system (ie, the degree to which a system is fit to be used) to ensure that the system is accessible, clear, and easy to use [21,22]. A final aspect of testing an intervention is to understand implementation processes and context, such as the acceptability and appropriateness. Feasibility, usability, and other implementation-related outcomes are all important to the translation of research into practice, providing better insights into service delivery [23]. The aim of this study was to test and evaluate the feasibility and usability through an implementation perspective of an internet-based pain education and exercise program for chronic musculoskeletal pain and to compare this program with a program consisting of an intervention with only a web-based self-management booklet.

## Methods

### Ethical Considerations and Study Design

This is a pilot study of a prospectively registered (NCT04274439), parallel, assessor-blinded, 2-arm randomized controlled trial with economic evaluation. This study was submitted and accepted by the ethics committee Comite de Ética em Pesquisa da Universidade Cruzeiro do Sul (CAAE 02892918.0.0000.8084), and the study protocol of the main trial has been published elsewhere [24].

### Settings and Eligibility

We recruited patients from the waiting lists of physical therapy and rehabilitation centers and through advertisements on social media networks. We included patients aged between 18 and 60 years, who were seeking treatment or who would like to undertake a physical therapy program for any chronic musculoskeletal pain, were able to read and understand Portuguese, and had internet access. We included patients with chronic musculoskeletal pain (pain lasting more than at least 12 weeks) [1] and pain intensity of at least 3 points on a 0-10 Numeric Pain Rating Scale [25]. We did not include patients who had nerve root compromise, serious pathologies (eg, fracture, tumor, inflammatory, autoimmune/infectious diseases), cardiovascular and metabolic diseases (eg, coronary heart disease, heart failure, decompensated diabetes), recent orthopedic surgery (in the last 12 months), surgery scheduled for the next 6 months, or were pregnant. Patients were also excluded if there was any contraindication to exercise measured with the Physical Activity Readiness Questionnaire Portuguese version [26,27].

### Procedures

The conduct of the study as well as the evaluations and 8-week follow-ups were carried out completely remotely through web-based platforms and telephone calls. We invited patients from waiting lists of rehabilitation centers via phone call, or participants seeking physiotherapy care could also contact the researchers of the study. After confirming eligibility of the participant, we scheduled the assessment session (baseline) through a videoconference. This session was performed using the platform Whereby to access an encrypted and personalized room with the patient and the researcher conducting the assessment. In this session, the researcher explained the study and all procedures as well as double checked the eligibility of the participant. Then, the participant received a consent form (signed electronically), completed the baseline assessment of the study, and finally received a login and password to access the study website [28] to be randomized automatically to one of the 2 groups at the first login.

### Outcome Measures

#### Primary Outcomes

All outcomes were measured after the intervention period (8 weeks) through an electronic form, with a study evaluator blinded to the treatment allocation. The primary outcomes were program fit (acceptability, appropriateness, and feasibility), system usability, societal costs, and feasibility of the main trial.

##### Program Fit

Program fit was measured using 3 measures composed of 4 items each that can be used independently or representing 1 single score [29]:

1. Acceptability of Intervention Measure (AIM): Acceptability is the perception among implementation stakeholders that a given treatment, service, practice, or innovation is agreeable, palatable, or satisfactory [29].
2. Intervention Appropriateness Measure (IAM): Appropriateness is the perceived fit, relevance, or compatibility of the innovation or evidence-based practice for a given practice setting, provider, or consumer, and perceived fit of the innovation to address a particular issue or problem [29].
3. Feasibility of Intervention Measure (FIM): Feasibility is defined as the extent to which a new treatment or an innovation can be successfully used or carried out within a given setting [29].

These measures are composed of 4 items, and the participant can answer as “totally agree,” “agree,” “neither agree nor disagree,” “disagree,” or “strongly disagree.” A value from 1 to 5 is assigned to each answer with a total value of 20 points possible for each measure. There are no cutoff points for these measures, but values closer to 5 in each answer and 20 in the total measure indicate better results for the proposed outcomes.

##### System Usability

To assess system usability, we used the System Usability Scale [30,31]. The System Usability Scale is composed of 10 items where the patients respond with “strongly disagree,” “disagree,” “neither agree nor disagree,” “agree,” or “strongly agree.” A value from 1 to 5 is assigned to each answer. The responses are summed and multiplied by 2 so that the total score ranges from 0 to 100 points, where scores closer to 100 indicate better usability results.

##### Societal Costs

We measured societal costs by the estimate of trial intervention costs, health care costs (visits to general practitioners, physiotherapists, alternative therapists, medical specialists, as well as the use of emergency, hospitalization, and medication), patients costs (transportation: the number of public transport tickets needed to get to the health care service), and lost productivity costs (absenteeism). Costs were measured based on the participants’ reported use of the resources by using a cost diary given to the participants at baseline. Participants were asked to send the completed cost diary to the evaluators or respond with the diary data in a web-based questionnaire. The health care costs were also divided into health insurance costs, public health system costs (*Sistema Único de Saúde*), and private costs (out-of-pocket costs). All costs were collected in BRL, inflated to the reference year of the study (2020) using the consumer price index and converted into USD using purchase power parities [32].

The intervention costs were estimated based on the total costs of website development, creation of the content included in the physical therapy program, internet hosting, and costs of text messages and phone calls present in each work group. The development and creation costs were considered from the perspective of using the program for over 5 years, based on the time of updating the information for the treatment in clinical practice guidelines. The value for 5 years of use was divided by the number of patients who would benefit from the treatment in each of the main clinical trial groups.

The unit prices of the health care services were calculated based on the Brazilian database (*Banco de Preços em Saúde*) [33] or from the Brazilian professional regulatory councils [34]. The unit prices of the medications that were not present in the Brazilian database were valued through a web-based commercial consultation in pharmacy chains (the unit average of these medications was calculated using the prices found in 5 pharmacy chains).

The transportation costs were estimated using the public transport price in the city of São Paulo, Brazil [35]. The lost productivity costs included absenteeism from paid work. Absenteeism was estimated by asking patients the number of hours not worked owing to chronic musculoskeletal pain and valued according to the Human Capital Approach using sex-specific price weights [36].

##### Feasibility of the Main Trial

The criteria to judge the feasibility of progressing to a full trial were as follows:

1. Fifty percent or more of the invited participants were willing to be recruited into the feasibility study.
2. Seventy percent or more of the participants in the intervention group have completed the 8-week program (compliance).
3. Data on key outcomes were collected at postintervention for ≥70% of participants.
4. Less than 10% of adverse events are caused by the intervention.

We also collected the response rate at follow-up and adherence to the program.

#### Secondary Outcomes

A key aim of the pilot study was to determine whether the primary and secondary outcomes for a proposed full trial could be measured for all the participants. This pilot study was not powered to detect significant differences in these measures but was able to describe observed changes between time points and their direction. Thus, the secondary outcomes of this study were pain intensity and function measured at baseline and postintervention (8 weeks) and adverse events measured during the intervention period.

1. Pain intensity was measured using the Numeric Pain Rating Scale [25], a numerical scale where 0 indicates no pain and 10 indicates the worst possible pain.
2. Function was measured with the Patient-Specific Functional Scale [25], a self-reported scale specific for the measurement of functionality, where the patients nominate up to 3 activities relevant to them and rate their ability to perform each activity on a 0 to 10 scale, with 0 representing the inability to perform that activity and 10 the total capacity to perform the activity. The sum total of the values for the 3 activities will be considered the final score on a 0 to 30 scale.
3. Adverse events: number and percentage of participants experiencing any adverse events during the intervention period (eg, exacerbation of symptoms).

### Random Allocation

The random allocation sequence was generated using computer software with a 50% chance of allocation for each of the groups. After the initial screening and baseline outcome assessment, patients were given a login and password to access the study site. As soon as the participant entered the site for the first time, they were randomly allocated to one of the 2 study groups.

### Blinding

The outcome assessor was blinded to the treatment groups. Owing to the nature of the interventions, it was not possible to blind the patients or the therapists.

### Interventions

#### Intervention Group: Internet-Based Pain Education and Exercise Program

The patients allocated to the intervention group received a login and password for individual access to the website developed for the study [28]. The content of this intervention included videos and animations based on pain education, promotion of physical activity, and general exercises. The pain education component was based on the e-pain intervention developed by Reis et al [12], which had 9 main features: (1) acceptance, (2 and 3) pain education, (4) sleep hygiene, (5) recognition of stress and negative emotions, (6) increased positive coping in lifestyle, (7) exercise, (8) communication, and (9) prevention. The exercise program was created by professional physiotherapists with at least 5 years of clinical experience, who are specialists in the treatment of chronic pain and who used exercise-based treatment. After its elaboration, it was submitted to a round of suggestions and adjustments to the program by a panel of experts. After this round of suggestions, the exercise component was sent by email to a group of experts who are references in the treatment of chronic pain in São Paulo, Brazil. After the last round of suggestions and corrections, the exercise program was modified to be simple and assertive for the population studied. The exercise component included general exercises with the aim of improving strength, flexibility, control, and coordination.

The total duration of the intervention was 8 weeks. There was new content every week of the intervention, and the patients were instructed to perform the video exercises at least 3 times a week and watch the videos as necessary. Patients in this group also received weekly text messages and a health coach over the phone. The text messages included information on the benefits of exercise and motivational and positive messages on how to deal with pain. The health coaching sessions were conducted once a week until the end of the intervention (8 weeks) by a physiotherapist with 5 years of experience and prior training for the coach’s performance. The goal of the health coach component is to keep patients motivated to continue with the program. This included encouragement, motivation, coping, revision of instructions and, if necessary, adaptation of the content of the intervention. For example, if a patient felt some discomfort when doing an exercise, the coach slightly modified the exercise (eg, dose, range of motion).

#### Control Group: Web-Based Self-management Booklet

Patients allocated to the control group had access to a web-based booklet containing general information on self-management of chronic pain, including pain education, advice on healthy lifestyle and sleeping habits, and promotion of physical activity. They also received a call in week 4 and motivational text messages once a week during the study period.

### Statistical Methods

#### Calculation of the Sample Size

For the pilot study, we determined a value of at least 40% of the total sample size of the main study. Our sample size calculation determines that a minimum of 160 individuals would be required for the main study analysis; therefore, at least 64 patients were necessary for the pilot phase.

#### Data Analysis

The normality of the data was tested by visual inspection of the histograms. The baseline characteristics of the participants were analyzed using descriptive statistics and summarized in a descriptive table. We consider the system’s usability greater than 53 points on a scale from 0 to 100 to be satisfactory in at least 70% of the responses observed. Primary and secondary outcomes were presented descriptively with means and standard deviations or number of participants and percentages for each group. Results were all presented in descriptive tables.

## Results

### Participant Characteristics

**Figure 1:**
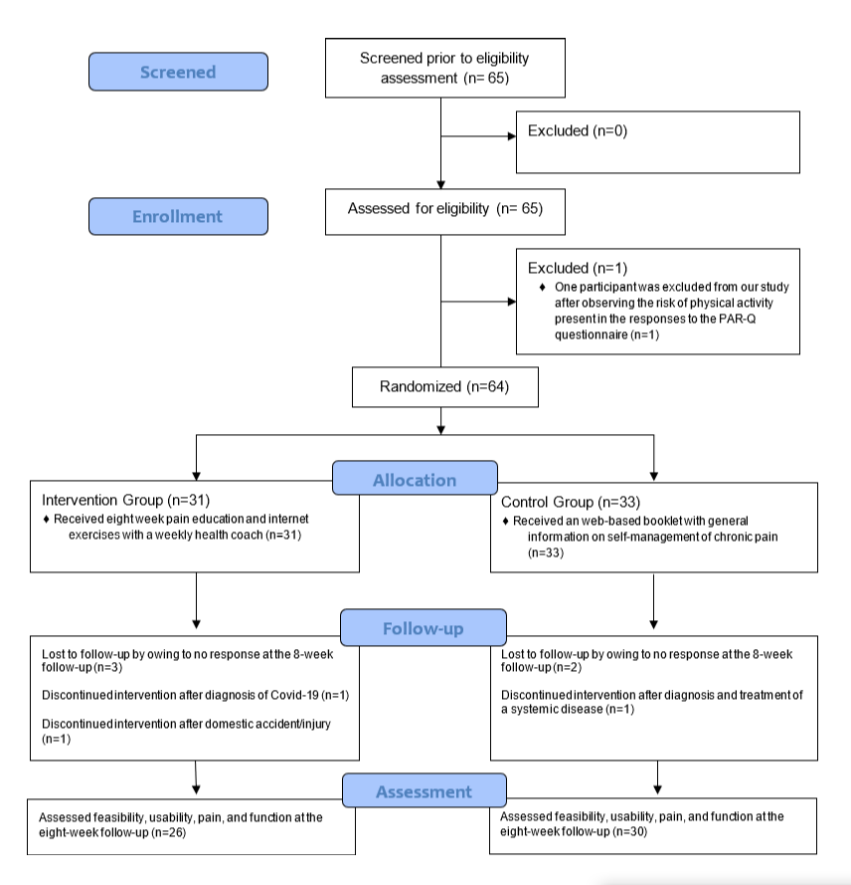
Flow diagram of recruitment. PAR-Q: Physical Activity Readiness Questionnaire.

A total of 65 participants were recruited between February 2020 and June 2020. Of these, 64 was randomized in the study. One participant was not randomized due to contraindication to exercise. A total of 31 participants were randomized to the intervention group and 33 participants to the control group (Figure 1). The participants were mostly women (43/64, 67%), with a mean age of 39.5 (SD 11.3) years, mean pain intensity of 6.3 (SD 1.6) points, and a median duration of symptoms of 36 (IQR 11-90) months. For the location of pain characteristics, patients were able to name more than one pain site. There was no difference between the groups at the baseline. A summary of the characteristics of the participants is shown in Table 1.

**Table 1:** Baseline characteristics of the participants (N=64).

Characteristics		Intervention group (n=31)	Control group (n=33)
Age (years), mean (SD)		40.2 (11.6)	38.8 (10.9)
Weight (kg), mean (SD)		77.1 (15.1)	76.6 (17.8)
Height (cm), mean (SD)		169.4 (8.6)	166.4 (7.1)
**Sex, n (%)**			
	Male	12 (39)	9 (27)
	Female	19 (61)	24 (73)
**Location of pain, n (%)**			
	Low back	16 (52)	19 (58)
	Cervical	5 (16)	8 (24)
	Dorsal	4 (13)	3 (9)
	Knee	9 (29)	8 (24)
	Shoulder	7 (23)	7 (21)
	Hip	5 (16)	7 (21)
	Ankle	2 (6)	4 (12)
	Elbow	2 (6)	3 (9)
	Hand/wrist	1 (3)	3 (9)
	Nonrespondent	4 (13)	7 (21)
**Education status, n (%)**			
	Primary education	4 (13)	0 (0)
	Secondary education	8 (26)	11 (33)
	Undergraduate	19 (61)	22 (67)
Physically active, n (%)		15 (48)	18 (55)
**Type of physical activity, n (%)**			
	Walking	3 (10)	4 (12)
	Strength exercises (gym)	9 (29)	9 (27)
	Stretching exercises	0 (0)	2 (6)
	Aerobics	1 (3)	1 (3)
	Running	1 (3)	1 (3)
	Functional training	1 (3)	0 (0)
	Pilates	1 (3)	2 (6)
	Dancing/ballet	3 (10)	4 (12)
	Cycling	0 (0)	1 (3)
	Football	2 (6)	2 (6)
	Swimming/hydro	1 (3)	1 (3)
	Volleyball	1 (3)	0 (0)
	Yoga	0 (0)	1 (3)
Medication use, n (%)		13 (42)	14 (42)
**Type of medication, n (%)**			
	Anti-inflammatory	4 (13)	6 (18)
	Analgesic	8 (26)	9 (27)
	Opioids	4 (13)	3 (9)
	Anticonvulsant	1 (3)	1 (3)
	Antidepressant	1 (3)	2 (6)
Pain duration (months), median (IQR)		36 (10-60)	36 (12-120)
Pain intensity (0-10), mean (SD)		6.0 (1.7)	6.5 (1.4)
Function (0-30), mean (SD)		18 (6.5)	17 (6.3)

### Adherence to the Trial Protocol

A total of 56 responses were obtained after the 8-week period of initiation of treatment in our study: 26 in the telerehabilitation group and 30 in the control group. Eight patients dropped out from the study because they did not answer our questionnaires on the correct date of follow-ups. For this pilot study, answers to questionnaires sent after the follow-up deadline were disregarded in order to calculate the possible sample loss. Adherence to the 8-week program (telerehabilitation group) was high, with a mean of 7.2 (SD 1.4) intervention weeks accessed by the patients on the program website. The weekly health coaching was successful in 92% (24/26) of the patients.

### Program Fit

All patients in the telerehabilitation group showed acceptable responses with satisfactory scores in all the responses for AIM, IAM, and FIM. Patients in the intervention group showed responses with a mean of 4.5 (SD 0.6) points for AIM, 4.5 (SD 0.5) points for IAM, and 4.5 (SD 0.6) points for FIM. For patients in the control group, satisfactory scores were found in 95% (114/120) of the responses for AIM, 95.8% (115/120) of the responses for IAM, and 99.2% (119/120) of the responses for FIM. Patients in the control group showed responses with a mean of 4.1 (SD 0.9) points for AIM, 4.1 (SD 0.8) points for IAM, and 4.3 (SD 0.6) points for FIM (Table 2).

**Table 2:** Description of the implementation outcomes of acceptability, appropriateness, feasibility, and usability.

Measures			Intervention group (n=26)		Control group (n=30)
**Acceptability of Intervention Measure, mean (SD)**					
	I approve the ReabilitaDOR program	4.68 (0.6)		4.3 (0.7)	
	ReabilitaDOR program is appealing to me	4.36 (0.7)		3.86 (1.0)	
	I like the ReabilitaDOR program	4.48 (0.6)		4.2 (0.8)	
	I welcome the ReabilitaDOR program	4.48 (0.7)		4.23 (0.8)	
	Total score	4.5 (0.6)		4.1 (0.9)	
**Intervention Appropriateness Measure, mean (SD)**					
	ReabilitaDOR program seems fitting	4.56 (0.5)		4.23 (0.8)	
	ReabilitaDOR program seems suitable	4.52 (0.5)		4.2 (0.8)	
	ReabilitaDOR program seems applicable	4.48 (0.5)		4.2 (0.7)	
	ReabilitaDOR program seems like a good match	4.6 (0.5)		4.13 (0.8)	
	Total score	4.5 (0.5)		4.1 (0.8)	
**Feasibility of Intervention Measure, mean (SD)**					
	ReabilitaDOR program seems implementable	4.62 (0.6)		4.26 (0.6)	
	ReabilitaDOR program seems possible	4.72 (0.5)		4.26 (0.7)	
	ReabilitaDOR program seems doable	4.56 (0.6)		4.33 (0.5)	
	ReabilitaDOR program seems easy to use	4.48 (0.7)		4.33 (0.6)	
	Total score	4.5 (0.7)		4.3 (0.6)	
**System Usability Scale**					
	Worst imaginable (0-20.5), n (%)	0 (0)		1 (3)	
	Poor (21-38.5), n (%)	0 (0)		0 (0)	
	Average (39-52.5), n (%)	0 (0)		2 (7)	
	Good (53-73.5), n (%)	3 (12)		12 (40)	
	Excellent (74-85.5), n (%)	7 (27)		11 (37)	
	Best imaginable (86-100), n (%)	16 (62)		4 (13)	
	Total score, mean (SD)	87 (10.7)		70 (17.9)	

### System Usability

All patients in the intervention group showed satisfactory responses to the usability outcome, with 16 patients responding as “best imaginable,” 7 responding as “excellent,” and 3 responding as “good” for all system features. The total mean score for usability in the intervention group was 87 (SD 10.7) points, classifying the usability by this group as “best imaginable.” In the control group, we observed 4 patients responding as “best imaginable,” 11 responding as “excellent,” 12 as “good,” 2 as “average,” and 1 responding as “worst imaginable.” The total mean score for usability in the control group was 70 (SD 17.9) points, classifying the usability by this group as “good” (Table 2).

### Societal Costs

A total of 55 responses were obtained from the cost diaries in the 8-week assessment. One patient in the control group did not respond to the cost diary; thus, the costs of this patient were not analyzed. The cost diaries of 29 patients in the control group and 26 patients in the intervention group during the 8-week period were analyzed. The intervention costs were US $210.60 per patient in the intervention group and US $20.62 per patient in the control group. The health care costs related to the private costs were US $54.17 per patient in the intervention group and US $107.23 per patient in the control group. For the public health system costs, we did not observe any cost in the intervention group, but we observed an expenditure of US $2.75 per patient in the control group. For the health insurance costs, we observed an expenditure of US $8.53 per patient in the intervention group and US $6.63 per patient in the control group. The total sum of individual private costs of the 2 groups presented an expenditure of US $62.73 per patient in the intervention group and US $116.63 in the control group. Regarding the transportation used by patients, the intervention group used 72 fares, totaling an expenditure of US $5.07 per patient. The control group used 68 fares, totaling an expenditure of US $4.29 per patient. The lost productivity costs for the intervention group were 3.3 hours per patient, totaling an expenditure of US $64.63 per patient and those for the control group were 15.5 hours per patient, totaling an expenditure of US $217.30 per patient. All cost values are shown in Table 3.

**Table 3:** Description of costs at 8-week follow-up.^a^

Costs		Intervention group (n=26)	Control group (n=29)
Intervention costs		$210.60	$20.62
**Health care costs**			
	Private costs	$54.10	$107.23
	Public costs	$0	$2.75
	Health insurance costs	$8.53	$6.63
	Total health care costs	$62.63	$116.61
**Patient costs**			
	Transport	$5.07	$4.29
**Absence from work**			
	Hours not worked	$64.63	$217.30
Total societal costs		$342.93	$358.82

^a^All costs are estimated in USD per patient.

### Feasibility of the Main Trial

The feasibility of the main trial was confirmed with regard to our prespecified criteria. A total of 98% (64/65) of the patients who were initially evaluated to enter the study were able to randomize and participate in the trials. A total of 20 patients (77%) of the 26 patients in the intervention group finished the 8-week program, 2 patients (8%) reached at least 6 weeks of the program, 2 patients (8%) reached 5 weeks of treatment, and 2 patients (8%) did not reach half of the treatment.

### Pain Intensity and Function

Both groups reported a decrease in pain intensity and improvement in function after 8 weeks. For the intervention group, we observed a mean of 6 (SD 1.8) points for pain intensity at the baseline and a mean of 3.4 (SD 2.4) points at the 8-week follow-up. In the control group, we observed a mean of 6.5 (SD 1.5) points for pain intensity at the baseline and a mean of 5.6 (SD 1.9) points at the 8-week follow-up. For function, in the intervention group, we observed a mean of 18 (SD 6.7) points at the baseline and a mean of 23 (SD 6.3) points at the 8-week follow-up. In the control group, we observed a mean of 17 (SD 6.4) points at the baseline and a mean of 20 (SD 5.5) points at the 8-week follow-up. No statistical inferential tests were conducted, as this was not the purpose of this pilot study. The means of pain intensity and function are shown in Table 4.

**Table 4:** Description of the secondary outcomes (pain intensity and function).

Outcome	Baseline		8-week follow-up	
	Intervention group (n=26)	Control group (n=30)	Intervention group (n=26)	Control group (n=30)
Pain intensity, mean (SD)^a^	6.0 (1.8)	6.5 (1.5)	3.4 (2.4)	5.6 (1.9)
Function, mean (SD)^b^	18 (6.7)	17 (6.4)	23 (6.3)	20 (5.5)

^a^Pain intensity is measured on a 0-10 scale; lower values mean less pain.

^b^Function is measured on a 0-30 scale; higher values mean greater function.

### Adverse Effects

No serious adverse effects were observed in carrying out the proposed interventions related to our study. One patient in the intervention group reported having been diagnosed with COVID-19 during the study. The same patient did not answer the posttreatment questionnaire. A patient in the intervention group reported a foot injury, resulting from the fall of a heavy object during a domestic task.

## Discussion

We found that the ReabilitaDOR program is feasible, appropriate, and acceptable from the users’ implementation perspective. This system has been considered usable by all the participants, and the main trial seems feasible. Costs and clinical outcomes were viable to be collected, the program was unlikely to cause harm, and no adverse events were reported during the intervention period. We had a low loss with follow-up and good levels of adherence and engagement with the health coach. The results of this study can demonstrate the feasibility of the main cost-effectiveness trial without major changes to the program.

Over the last few decades and in the past years of the COVID-19 pandemic, we have observed an increase in studies involving telerehabilitation platforms for patients with chronic pain [17]. However, it is important to emphasize that the intervention implementation processes are dependent on the context, cultural diversity, and characteristics of the population [37,38]. Thus, this study is innovative and novel because it included a population that was never been exposed to telerehabilitation before. Brazil incorporated the first regulation on telehealth in March 2020 after the social isolation policies imposed by the COVID-19 pandemic. These results may pave the way for new initiatives involving telehealth in low- and middle-income countries, since previous studies with this proposal were predominantly carried out in high-income countries [39].

The observation of results in low- and middle-income countries is necessary and meets the needs of care and equity provided by the World Health Organization [14,39]. To reach that, it is extremely important to design studies that prioritize the analysis of outcomes that are related to the implementation process [23,40]. Implementation science can help reduce the actual implementation time of interventions and provide many benefits to science and clinical practice. Thus, the writing of this study followed the recommendations of the CONSORT statement for feasibility and pilot studies [41] and is based on a concrete proposal on how to use implementation science and design studies with this objective, thereby being a differential among several pilot studies [20,42]. Although we were unable to carry out an intervention mapping process, we followed the observation of the outcomes such as acceptability, adoption, appropriateness, feasibility, program fidelity, and costs [23,40,43].

The future implications of the results observed in this study are toward a better understanding of the processes of use of telerehabilitation in countries that do not have a regulation for its use or have a recent regulation. Our study explores the results of a sample that has never been exposed with the type of intervention that was performed, being extremely important for the real visualization of the implementation outcomes in populations that would benefit from telerehabilitation but for whatever reason did not have this experience. In addition to the clinical implications, this study aims to test the feasibility of the main study, which will provide specific data on the effectiveness and cost-effectiveness of the tested program. Conducting a pilot study with a representative sample can provide insights into the future implementation and feasibility results [44] and should be encouraged, as many pilot studies do not demonstrate outcomes inherent to their role [42].

Our study has some limitations that may be important for the general interpretation of the results. The 3 implementation outcomes tested were initially proposed to measure the acceptability, adoption, and feasibility of clinicians and stakeholders in implementing evidence-based practices [29]. In our study, implementation outcomes were not collected from the stakeholders’ perspective; instead, we approached the patients directly. We believe this is also important because asynchronous telerehabilitation strategies are dependent on patients’ adherence, acceptance, appropriateness, usability of the system, and willingness to navigate the platform [23,40]. Our research group is also investigating users’ barriers and facilitators through qualitative methodologies within the main clinical trial. The arrival of SARS-CoV-19 in Brazil and the declaration of a pandemic by the World Health Organization brought the emergency need for measures to contain the disease, which included social distancing and quarantine [45]. In the period in which we began to recruit patients on the waiting lists of physiotherapy clinics, we faced a drastic decrease in the search for physiotherapy and health care services [46]. Thus, recruitment was carried out primarily through social media, giving the profile of our sample a specific characteristic of patients who already used the internet.

In conclusion, we found that the ReabilitaDOR program is feasible, appropriate, and acceptable from the users’ implementation perspective. This system has been considered usable by all the participants, and the main trial seems feasible. Cost data were viable to be collected and the program is unlikely to cause harm, as no adverse events were reported during the intervention period. Both groups reported being overall satisfied with the platform and the proposed program content.

## Abbreviations

AIM: Acceptability of Intervention Measure
FIM: Feasibility of Intervention Measure
IAM: Intervention Appropriateness Measure

## References

[1] Treede R, Rief W, Barke A, Aziz Q, Bennett M, Benoliel R, Cohen M, Evers S, Finnerup N, First Michael B, Giamberardino Maria Adele, Kaasa Stein, Kosek Eva, Lavand'homme Patricia, Nicholas Michael, Perrot Serge, Scholz Joachim, Schug Stephan, Smith Blair H, Svensson Peter, Vlaeyen Johan W S, Wang Shuu-Jiun (2015). A classification of chronic pain for ICD-11. Pain.

[2] Harvey AM (1995). Classification of Chronic Pain—Descriptions of Chronic Pain Syndromes and Definitions of Pain Terms. The Clinical Journal of Pain.

[3] Carvalho R, Maglioni C, Machado G (2018). Araújo JEd, Silva JRTd, Silva MLd: Prevalence and characteristics of chronic pain in Brazil: a national internet-based survey study. Brazilian Journal Of Pain 2018.

[4] Marinho F, de Azeredo Passos Vm, Carvalho Malta D, Barboza França E, Abreu Dmx, Araújo Vem, Bustamante-Teixeira Mt, Camargos Pam, da Cunha Cc, Duncan Bb, Felisbino-Mendes Ms, Guerra Mr, Guimaraes Mdc, Lotufo Pa, Marcenes W, Oliveira Ppv, de Moares Pedroso M, Ribeiro Al, Schmidt Mi, Teixeira Ra, Vasconcelos Amn, Barreto Ml, Bensenor Im, Brant Lcc, Claro Rm, Costa Pereira A, Cousin E, Curado Mp, dos Santos Kpb, Faro A, Ferri Cp, Furtado Jm, Gall J, Glenn Sd, Goulart Ac, Ishitani Lh, Kieling C, Ladeira Rm, Machado Ie, Martins Sco, Martins-Melo Fr, Melo Aps, Miller-Petrie Mk, Mooney Md, Nunes Bp, Palone Mrt, Pereira Cc, Rasella D, Ray Se, Roever L, de Freitas Saldanha R, Santos Is, Schneider Ijc, Santos Silva Da, Silveira Dga, Soares Filho Am, Moraes Sousa Tc, Szwarcwald Cl, Traebert J, Velasquez-Melendez G, Wang Y, Lozano R, Murray Cjl, Naghavi M (2018). Burden of disease in Brazil, 1990–2016: a systematic subnational analysis for the Global Burden of Disease Study 2016. The Lancet.

[5] GBD 2019 DiseasesInjuries Collaborators (2020). Global burden of 369 diseases and injuries in 204 countries and territories, 1990-2019: a systematic analysis for the Global Burden of Disease Study 2019. Lancet.

[6] Phillips CJ (2009). The Cost and Burden of Chronic Pain. Rev Pain.

[7] de Oliveira RF, Fandim JV, Fioratti I, Fernandes LG, Saragiotto BT, Pena Costa LO (2019). The contemporary management of nonspecific lower back pain. Pain Manag.

[8] Woby S, Roach N, Urmston M, Watson Paul J (2007). The relation between cognitive factors and levels of pain and disability in chronic low back pain patients presenting for physiotherapy. Eur J Pain.

[9] Lotze M, Moseley G Lorimer (2015). Theoretical Considerations for Chronic Pain Rehabilitation. Phys Ther.

[10] Stewart M, Loftus S (2018). Sticks and Stones: The Impact of Language in Musculoskeletal Rehabilitation. J Orthop Sports Phys Ther.

[11] Truchon M (2001). Determinants of chronic disability related to low back pain: towards an integrative biopsychosocial model. Disabil Rehabil.

[12] Reis FJ, Bengaly AG, Valentim JC, Santos LC, Martins EF, O'Keeffe Mary, Meziat-Filho N, Nogueira LC (2017). An E-Pain intervention to spread modern pain education in Brazil. Braz J Phys Ther.

[13] Saragiotto BT, Fioratti I, Tiedemann A, Hancock MJ, Yamato TP, Wang SS, Chau JY, Lin CC (2020). The Effectiveness of Strategies to Promote Walking in People With Musculoskeletal Disorders: A Systematic Review With Meta-analysis. J Orthop Sports Phys Ther.

[14] WHO Global Observatory for eHealth (2010). Telemedicine: opportunities and developments in member states: report on the second global survey on eHealth. WHO Global Observatory for eHealth.

[15] Tuckson RV, Edmunds M, Hodgkins ML (2017). Telehealth. N Engl J Med.

[16] Bashshur R, Shannon G, Krupinski E, Grigsby J (2011). The taxonomy of telemedicine. Telemed J E Health.

[17] Alan Lee, Karen Finnin, Lesley Holdsworth, Dianne Millette, Chris Peterson, Digital Physical Therapy Task Force (2019). Report of the world physiotherapy/INPTRA digital physical therapy practice task force. World Confederation for Physiotherapy.

[18] Leon AC, Davis LL, Kraemer HC (2011). The role and interpretation of pilot studies in clinical research. J Psychiatr Res.

[19] van Teijlingen E, Hundley V (2002). The importance of pilot studies. Nurs Stand.

[20] Story DA, Leslie K, French C (2018). Feasibility and Pilot Studies: Small Steps before Giant Leaps. Anaesth Intensive Care.

[21] Klaassen B, van Beijnum B, Hermens H (2016). Usability in telemedicine systems-A literature survey. Int J Med Inform.

[22] Fornazin M, Joia LA (2015). Articulando perspectivas teóricas para analisar a informática em saúde no Brasil. Saude soc.

[23] Proctor E, Silmere H, Raghavan R, Hovmand P, Aarons G, Bunger A, Griffey R, Hensley M (2011). Outcomes for implementation research: conceptual distinctions, measurement challenges, and research agenda. Adm Policy Ment Health.

[24] Fioratti I, Saragiotto BT, Reis FJJ, Miyamoto GC, Lee H, Yamato TP, Fandim JV, Dear B, Maher CG, Costa LOP (2020). Evaluation of the efficacy of an internet-based pain education and exercise program for chronic musculoskeletal pain in comparison with online self-management booklet: a protocol of a randomised controlled trial with assessor-blinded, 12-month follow-up, and economic evaluation. BMC Musculoskelet Disord.

[25] Costa LOP, Maher CG, Latimer J, Ferreira PH, Ferreira ML, Pozzi GC, Freitas LMA (2008). Clinimetric Testing of Three Self-report Outcome Measures for Low Back Pain Patients in Brazil. Spine.

[26] Andreazzi IM, Takenaka VS, Silva PSBD, Araújo MPD (2016). Exame pré-participação esportiva e o par-q, em praticantes de academias. Rev Bras Med Esporte.

[27] Thomas S, Reading J, Shephard RJ (1992). Revision of the Physical Activity Readiness Questionnaire (PAR-Q). Can J Sport Sci.

[28] ReabilitaDOR Program.

[29] Weiner BJ, Lewis CC, Stanick C, Powell BJ, Dorsey CN, Clary AS, Boynton MH, Halko H (2017). Psychometric assessment of three newly developed implementation outcome measures. Implement Sci.

[30] Martins AI, Rosa AF, Queirós A, Silva A, Rocha NP (2015). European Portuguese Validation of the System Usability Scale (SUS). Procedia Computer Science.

[31] Padrini-Andrade L, Balda RDCX, Areco KCN, Bandiera-Paiva P, Nunes MDV, Marba STM, Carvalho WBD, Rugolo LMSDS, Almeida JHCD, Procianoy RS, Duarte JLMB, Rego MAS, Ferreira DMDLM, Alves Filho N, Guinsburg R, Diniz EMDA, Santos JPFD, Testoni D, Silva NMDME, Gonzales MRC, Silva RVCD, Meneses J, Gonçalves-Ferri Walusa Assad, Perussi-E-Silva Ricardo, Bomfim O (2019). Evaluation of usability of a neonatal health information system according to the user's perception. Rev Paul Pediatr.

[32] OECD (2012). Eurostat-OECD Methodological Manual on Purchasing Power Parities.

[33] Banco de Preços em Saúde.

[34] (2020). Referência nacional de procedimentos fisioterapêuticos. Conselho Federal de Fisioterapia e Terapia Ocupacional.

[35] (2020). Valor das tarifas de transporte urbano da cidade de São Paulo. Secretaria Municipal de Transporte e Mobilidade Urbana.

[36] Miyamoto GC, Bosmans JE, van Tulder MW, Lin CC, Cabral CMN, van Dongen JM, Ben (2021). Interpretation of trial-based economic evaluations of musculoskeletal physical therapy interventions. Braz J Phys Ther.

[37] Cabassa LJ, Baumann AA (2013). A two-way street: bridging implementation science and cultural adaptations of mental health treatments. Implement Sci.

[38] Kagawa Singer M (2012). Applying the concept of culture to reduce health disparities through health behavior research. Prev Med.

[39] Reis FJ, Fernandes LG, Saragiotto BT (2021). Telehealth in low- and middle-income countries: Bridging the gap or exposing health disparities?. Health Policy Technol.

[40] Wolfenden L, Foy R, Presseau J, Grimshaw JM, Ivers NM, Powell BJ, Taljaard M, Wiggers J, Sutherland R, Nathan N, Williams CM, Kingsland M, Milat A, Hodder RK, Yoong SL (2021). Designing and undertaking randomised implementation trials: guide for researchers. BMJ.

[41] Eldridge SM, Chan CL, Campbell MJ, Bond CM, Hopewell S, Thabane L, Lancaster GA, PAFS consensus group (2016). CONSORT 2010 statement: extension to randomised pilot and feasibility trials. BMJ.

[42] Loscalzo J (2009). Pilot Trials in Clinical Research. Circulation.

[43] Glasgow RE, Harden SM, Gaglio B, Rabin B, Smith ML, Porter GC, Ory MG, Estabrooks PA (2019). RE-AIM Planning and Evaluation Framework: Adapting to New Science and Practice With a 20-Year Review. Front Public Health.

[44] Hertzog MA (2008). Considerations in determining sample size for pilot studies. Res Nurs Health.

[45] (2021). COVID-19 strategic preparedness and response plan: operational planning guideline: 1 February 2021 to 31 January 2022. World Health Organization.

[46] Miller G (2020). Social distancing prevents infections, but it can have unintended consequences. Science.

